# Editorial: Glimpse to the past – the evolution of the role of Imaging in cardio therapeutics

**DOI:** 10.3389/fcvm.2026.1900003

**Published:** 2026-07-08

**Authors:** Eren Ozan Bakir, Stefanie M. Werhahn, Ameer Hamid A. Khan, Christos V. Bourantas, Sebastian Kelle

**Affiliations:** 1 Centre for Cardiovascular Medicine and Devices, William Harvey Research Institute, Queen Mary University of London, London, United Kingdom; 2 Department of Cardiology, Bakircay University Cigli Training and Research Hospital, Izmir, Türkiye; 3 Department of Cardiology, Angiology and Intensive Care Medicine, Deutsches Herzzentrum der Charité, Berlin, Germany; 4 Department of Cardiology, Barts Heart Centre, Barts Health NHS Trust, London, United Kingdom; 5 German Centre for Cardiovascular Research (DZHK), Berlin, Germany

**Keywords:** CTA (computed tomographic angiography), IVUS (intravascular ultrasound), magnetic resonance imaging (MRI), OCT (optical coherence tomography), positron emission tomography - computed tomography (PET-CT)

Over the last century there is a dramatic change in the diagnosis and treatment of patients with cardiovascular diseases. The bedside clinical examination and physician's judgment that constituted the basis for diagnosis of cardiac pathologies has been replaced by advanced diagnostic investigations that include blood biomarkers and imaging tests which enables detailed visualization of the structure of the heart and main vessels and optimal treatment planning. The self-catheterization of Werner Forssmann's in 1929 provided proofs that the imaging of the heart and great vessels is possible *in vivo* and opened new horizons in cardiac diagnostics (1). Since then, advances in signal processing and imaging technologies have resulted in several invasive and non-invasive imaging techniques that allowed detailed evaluation of the coronary arteries and the myocardium, characterisation of their structure and quantification of their function. These developments not only allowed accurate diagnosis but have also opened new frontiers for therapeutic interventions that transformed the management of patients with cardiovascular disease. This special issue of Frontiers of Cardiovascular Medicine provides an overview of the evolution of cardiac imaging, it describes the first modalities introduced for the assessment of cardiac structures and their validation, challenges in the analysis and interpretation of the collected images methodologies that were developed for the processing of the acquired data and presents artificial intelligence algorithms designed for the fast analysis of large imaging sets.

Angiography has been the first modality that was used to assess cardiac structures. After the first coronary angiogram that was performed accidentally by Mason Sones coronary angiography has become the reference standard for assessing the extent and severity of coronary artery disease (2). The introduction of computerised-based methodologies for accurate and reproducible quantification of coronary stenosis has rendered it the ideal modality in the early days of percutaneous coronary intervention (PCI) for assessing the performance of interventional therapies, while in more recent years these approaches provided the substrate for the reconstruction of coronary artery anatomy and the computation of the fractional flow reserve and coronary microvascular resistance using either computational fluid dynamic techniques or dedicate formulas. The developments in the field are described by Gurav et al. who also summarised the clinical studies underscoring its role in PCI planning. Moreover, this review synopsises the evolution of computed tomography coronary angiography (CTA) as an alternative non-invasive tool for assessing coronary artery pathology and presents the methodologies that were introduced to assess lesion severity in CTA and guide PCI. The work of Gao et al. provides additional insights on the potential of CTA in the study of atherosclerosis and discusses the challenges in characterising plaque composition. It presents the studies showing an effect of different CTA systems, acquisition protocols and reconstruction methodologies on the quantification of different tissue types, based on established Hounsfield units cutoffs, and summarises the approaches that have been proposed to overcome this pitfall and the findings of their validation with a final aim to highlight the need to standardise image acquisition protocols and reconstruction methods and develop advanced AI-based solutions for accurate quantification of different tissue components.

AI-based CT image analysis appears also promising in peripheral interventions. The review of Xu et al. provides a comprehensive overview of the AI solutions that have been developed for the detection and treatment planning of aortic disease. It presents the approaches that have been proposed to enhance image quality, increase signal-to-noise and contrast-to-noise ratios and reduce radiation and contrast exposure without compromising diagnostic performance, it describes the methodologies proposed to expedite CT data analysis and accurately segment intimal flap, and summarizes the AI-based prognostication tools proposed for risk stratification particularly post endovascular aortic repair (EVAR). Moreover, the authors discuss the barriers that have limited their clinical application in routine workflows namely the limited data used for training, the differences in the CT imaging protocols and the anatomical complexity of aortic diseases and that have to be addressed so as to unlock their full potential in the treatment of aortic diseases.

A limitation of CTA in the study of atherosclerosis is its inability to assess plaque biology and inflammation. Positron emission tomography (PET) combined with CTA seems able to phenotype atherosclerotic activity and potentially detect more accurately high-risk plaques. The review of Bajaj et al. focuses on the value of PET imaging in the study of coronary atherosclerosis. It presents the established tracers developed to assess plaque inflammation and the evidence from studies examining their efficacy to detect high-risk lesions and patients. Moreover, the authors discuss the constraints of PET imaging and in particular the poor image resolution and the challenges in the co-registration of CTA and PET data and the solutions that were proposed to address these, and list emerging agents targeting molecular pathways that are expected to provide novel biological readouts of plaque inflammation.

Intravascular imaging modalities have played a pivotal role in the understanding of atherosclerotic evolution and in the maturation of interventional cardiology as they enabled *in vivo* visualization of coronary artery pathology and the effect of invasive therapies on vessel wall. The review of Abusnina et al. provides a comprehensive summary of the evolution in intravascular imaging. It describes the first prototype developed by Inge Edler and Carl Hertz in 1954 that used ultrasound to visualize cardiac structures and the efforts that were made over subsequent years to miniaturise intravascular imaging probes for *in vivo* invasive imaging, and presents the pioneer work of Adolf Fercher that demonstrated the potential of optical coherence tomography (OCT) in visualising eye fundus and the technical developments of Massachusetts Institute of Technology that allowed *in vivo* coronary imaging using OCT (3, 4). Finaly, the authors summarise the modalities that were introduced for an *in vivo* assessment of plaque biochemical composition and inflammation focusing mainly on the developments in near-infrared spectroscopy (NIRS) and give an overview of the field of hybrid imaging by describing the prototypes that combined multiple imaging probes with complementary strengths for more detailed characterization of plaque morphology and biology.

Additional insights about the value of intravascular imaging in characterising atheroma phenotypes are provided by Yap et al. who provided a detailed overview of the studies comparing the estimations of standalone catheters (intravascular ultrasound, OCT, NIRS, near infrared fluorescence, fluorescence lifetime imaging and intravascular photoacoustic imaging) against histology. The authors concluded that none of the existing systems are capable to provide a complete and detailed characterisation of plaque morphology and biology and underscored the need to design and introduce in the clinical practice hybrid intravascular imaging catheters that will have multiple probes acquiring co-registered imaging data and develop AI methods that will be trained against large histological datasets with multiple stains for fast accurate and reproducible quantification of plaque components.

Magnetic resonance imaging (MRI) constitutes today an indispensable tool for the diagnosis and treatment of cardiac diseases and constitutes the reference standard for the evaluation of heart structure and function. The manuscript of Sanghvi et al. presents the history of MRI, starting from the pioneer work of Felix Bloch and Edwin Purcell in 1946 that described the phenomenon of nuclear resonance imaging and of Peter Mansfield and Andrew Maudsley who provided proofs of the feasibility of this modality to assess tissue types and continuing with efforts made in recent years to overcome the challenges of cardiac motion and accurately quantify not only cardiac function but also characterise myocardial pathology using contrast agents and mapping protocols (5–7). The above developments and cumulative data highlighting the role of MRI in the diagnosis of cardiac disorders and risk stratification, led to the conduction of large population studies such as the Multi-Ethnic Study of Atherosclerosis and the UK Biobank study that have listed thousands of patients to cardiac MRI to examine the value of this modality in the early diagnosis and prediction of cardiovascular diseases (8, 9).

The wide applications of MRI in clinical practice and cardiovascular research created the need to develop automated solutions for the fast and reproducible analysis of the collected images. Several AI methods have been developed for this purpose; one of these is presented in this special issue by Salehi et al. who trained a U-net convolutional neural network for the segmentation of the left and right ventricle using the annotations of experts in 611 MRI scans as a reference standard. Testing in 421 MRI scans showed that the proposed method was fast and provided estimations about the left and right ventricular dimensions that correlated closely with the output of experts analysts. These findings are in line with previous studies in the field and indicate that AI-based tool can be used in clinical practice to expedite MRI reporting.

In conclusion, the reports included in this special issue of Frontiers Cardiovascular Medicine not only provide an overview of the evolution of cardiac imaging but also discuss the advantages and limitations of the existing modalities, their current applications in the clinical practice and research and the challenges that need to be addressed by novel imaging systems or efficient AI-based analysis methods so as to unlock their full potential in risk stratification, diagnosis and management of cardiac disorders.
